# Acquittal of Dr. Warren C. Westlake

**Published:** 1877-06

**Authors:** 


*Acquittal of Dr. Westlake.*—The jury in the case of Dr. Warren C. Westlake, of Elizabeth, New Jersey, charged with manslaughter, in causing the death of Walter Lewis with chloroform, while extracting a tooth, rendered a verdict of not guilty.

